# Detection and Phylogenetic Characterization of Canine Distemper Virus from a Red Fox in Hungary

**DOI:** 10.3390/v18030352

**Published:** 2026-03-13

**Authors:** Dominik Szieber, Ágota Ábrahám, Krisztián Bányai, Péter Malik, Alexandra Nándori, Brigitta Fézer, Árpád Bacsadi, Kornélia Bodó, Anna Szabó, Gábor Kemenesi, Zsófia Lanszki

**Affiliations:** 1 Institute of Biology, Faculty of Sciences, University of Pécs, 7624 Pécs, Hungary; szieber.dominik@edu.pte.hu (D.S.); abraham.agota@pte.hu (Á.Á.); szabo.anna@pte.hu (A.S.); kemenesi.gabor@pte.hu (G.K.); 2 National Laboratory of Virology, Szentágothai Research Centre, University of Pécs, 7624 Pécs, Hungary; bodo.kornelia@pte.hu; 3 Department of Medical Biology, Medical School, University of Pécs, 7624 Pécs, Hungary; bkrota@hotmail.com; 4 Department of Pharmacology and Toxicology, University of Veterinary Medicine, 1078 Budapest, Hungary; 5 Department of Virology, Directorate of the Veterinary Diagnostic Laboratory, National Food Chain Safety Office, 1143 Budapest, Hungary; malikp@nebih.gov.hu (P.M.); nandoria@nebih.gov.hu (A.N.); fezerb@nebih.gov.hu (B.F.); bacsadia@nebih.gov.hu (Á.B.)

**Keywords:** *Paramyxoviridae*, *Morbillivirus*, *Morbillivirus canis*, canine distemper virus, amplicon sequencing, NGS, MinION, red fox, disease ecology, integrated monitoring

## Abstract

Canine distemper virus (CDV) affects both domestic and wild carnivores and is associated with a high mortality rate. The virus can cross species barriers, infecting a wide range of mammals, which raises concerns for both wildlife conservation and domestic animal health. During our study, we processed a total of *n* = 552 oral and rectal swab samples from *n* = 260 red foxes (*Vulpes vulpes*) and *n* = 16 golden jackals (*Canis aureus*). The samples were collected by the National Food Chain Safety Office (NÉBIH) as part of a Rabies monitoring programme from Hungary in 2024. We performed a Real-Time RT-PCR, followed by a CDV-specific amplicon-based sequencing method using Oxford Nanopore Technologies to obtain the complete genome. All golden jackal samples tested negative, while both oral and rectal samples of one red fox tested positive for viral RNA. From this positive sample, we were able to sequence a partial CDV genome. Based on phylogenetic analysis of the haemagglutinin gene, our CDV sequence was assigned to the Europe lineage, one of the endemic lineages in the continent, infecting both threatened and common animals. This finding highlights the ongoing presence of CDV in wildlife populations and illustrates the value of integrated monitoring systems.

## 1. Introduction

Monitoring and surveillance programmes play a key role in the effective conservation of biodiversity and the mitigation of risks to human health, as the emergence of infectious diseases occurs worldwide [1,2], driven by habitat loss and increased interactions between wildlife and humans [3]. As a consequence of increasing global interactions, a growing number of infectious diseases are shared between domestic and wild animals, posing a threat to the economy and human health [4]. Canine distemper virus (CDV) is a prototype pathogen representing a multi-host, globally distributed and genetically highly adaptive virus [5]. Therefore, monitoring programmes provide valuable insights into virus circulation and emergence, leading to a better understanding of CDV epidemiology. Integrated monitoring represents a crucial component of surveillance systems, as it targets multiple pathogens within the same potential host population or utilizes existing monitoring systems for multiple purposes [2,6]. These systems combine molecular pathogen screening and genomic characterization with existing wildlife health monitoring activities, rather than operating as separate pathogen-specific programmes. This structure enables the assessment of multiple infectious agents from the same specimens, improving efficiency and coordination in wildlife disease surveillance. Monitoring programmes may help prevent future outbreaks, as early detection of pathogens enables the implementation of countermeasures, including vaccination campaigns similar to those used for rabies [7].

CDV is a negative-sense single-stranded RNA virus of the *Paramyxoviridae* family in the *Morbillivirus* genus [8,9]. Multiple CDV strains have been identified worldwide, based on the sequence of the Hemagglutinin (H) gene, which encodes the H protein that plays a key role in viral entry and is associated with the occurrence of the disease [10]. Although CDV strains can be identified based on the sequence of the H gene, complete genome sequencing provides deeper insight into the evolutionary dynamics and molecular mechanisms of the virus [10]. CDV is well known for infecting a wide range of both domestic and wild animal species [11,12,13,14,15,16], and its transmission is associated with a high likelihood of cross-species infection [17,18]. This is an already-known threat to animal health and wildlife conservation. There are several documented cases in the literature where endangered and vulnerable species across various families were affected by the virus [11,12,19,20,21].

From a local perspective, the presence of CDV has been confirmed among several members of the *Mustelidae* family. These species are well known to be highly susceptible to CDV, and infections often lead to an almost 100% mortality rate [22,23]. Such events have been documented in Hungary and throughout Europe [14,19,24]. CDV is a ubiquitous pathogen infecting several *Canid* species such as red foxes (*Vulpes vulpes*), golden jackals (*Canis aureus*), and domestic dogs *(Canis lupus familiaris*). Several cases have been reported in Hungary among red foxes and domestic dogs, including a single outbreak in 2021 among wild red foxes caused by the Europe lineage of CDV [25]. This lineage is known to circulate across Europe among various species of carnivorous mammals [5,13,14,16,26,27]. The other two lineages described from Hungary are the European-wildlife lineage, which had not been detected in the region since 2006 [28], and the Arctic-like lineage, which was detected in a domestic dog in 2019 and resulted in PCR positivity persisting for 17 months [15].

Next-generation sequencing (NGS) technologies play a key role in the characterization of a wide range of pathogens, as these technologies allow rapid sample preparation and can be optimized for specific targets. Oxford Nanopore Technologies, a long-read sequencing platform, enabled real-time sequence analysis, allowing the immediate assessment of sequencing data [5,6,28,29]. The amplicon-based NGS approach employed in our study is well-suited for amplifying samples with low concentrations of viral nucleic acids to generate complete or near-complete viral genome sequences [25,30,31,32].

The detection and characterization of infectious diseases plays a crucial role in wildlife conservation, outbreak prevention, and understanding transmission and pathogenesis. Surveillance of non-protected species via hunting programmes provides an efficient strategy to monitor circulating pathogens in wildlife and gives a stable background for integrated surveillance strategies. The aim of this study was to investigate the presence of CDV in red foxes and golden jackals, and to characterize the virus through genome sequencing and phylogenetic analysis.

## 2. Materials and Methods

### 2.1. Sample Collection

Oral and rectal swab samples were collected from red foxes *n* = 260 and golden jackals *n* = 16, leading to a total number of 552 samples. The sampling was organized and conducted by the National Food Chain Safety Office (NÉBIH) within the framework of the Rabies Monitoring Program in Hungary, with samples collected from five counties located in the eastern part of the country (Table 1). For each carcass, separate sterile swabs and collection tubes were used to avoid cross-contamination. The sampling was conducted between 1 May and 1 August 2024. All samples originated from adult animals that showed no apparent clinical signs at the time of collection.

### 2.2. Nucleic Acid Extraction and PCR Reactions

The samples were homogenized in 300 µL of phosphate-buffered saline (PBS), from which 100 µL of the supernatant was used for nucleic acid extraction using the Quick-RNA Miniprep Kit (Zymo Research, Irvine, CA, USA). All nucleic acid samples were tested by a CDV-specific Real-Time RT-PCR assay to detect the presence of viral RNA [33]. The Luna^®^ Universal One-Step RT-qPCR Kit (New England Biolabs, Ipswich, MA, USA) was used for PCR reactions. The amplification protocol consisted of reverse transcription at 55 °C for 10 min, initial denaturation at 95 °C for 1 min, followed by 45 cycles of denaturation at 95 °C for 10 s, annealing at 46 °C for 15 s, and elongation at 60 °C for 15 s (the probe was labelled with FAM, and fluorescence signal was detected during the annealing step). The test was run on the MyGo Mini PCR system platform (IT-IS Life Science, Dublin, Ireland) and analysed with the MyGo PCR software (v.3.5.2) with Ct values determined automatically. Each sample was run in duplicate with appropriate positive and negative controls. RT-PCRs were performed immediately after RNA extraction without freeze–thawing the nucleic acid, avoiding RNA degradation.

### 2.3. Nanopore Sequencing and Bioinformatics Analysis

MinION platform (Oxford Nanopore Technologies, Oxford, UK) was used for complete genome sequencing, using a slightly modified protocol developed in our laboratory [25,32]. Superscript IV Reverse Transcriptase (Invitrogen, Waltham, MA, USA) was used together with random hexamers to synthesize cDNA from positive samples. Q5 Hot Start High-Fidelity Polymerase (New England Biolabs, Ipswich, MA, USA) was used with multiple primer sets to generate overlapping amplicons (size range, approx. 1000 and 2000 nt [25,32]) from the previously prepared cDNA. End-repair was carried out using the NEBNext Ultra II End Repair Module (New England Biolabs, Ipswich, MA, USA). Following the end-repair, library preparation was carried out using the SQK-NBD114.96 Native Barcoding Kit (Oxford Nanopore Technologies, Oxford, UK) and the recommended 3rd-party materials. Subsequently, the final library concentration was measured with a Qubit fluorimeter using the Qubit dsDNA HS Assay Kit (Invitrogen, Waltham, MA, USA), and the prepared library was finally loaded onto an R10.4.1 flow cell on the MinION Mk1B device (Oxford Nanopore Technologies, Oxford, UK).

Basecalling and demultiplexing were performed on Ubuntu Linux 22.04 (Canonical, UK) using ONT Guppy software (version 6.5.7). Basecalling was conducted with Guppy basecaller (version 6.5.7, Oxford Nanopore Technologies, Oxford, UK) using the super-accurate basecaller algorithm (dna_r10.4.1_e8.2_400bps_sup.config file). Demultiplexing and barcode trimming were performed with Guppy barcoder (version 6.5.7). Sequence quality was assessed using NanoPlot (version 1.41). Demultiplexed reads were filtered by length with NanoFilt (version 2.0.8); reads from the 1000 nt amplicons were filtered to exclude sequences shorter than 300 bp and reads from the 2000 nt amplicons were filtered to exclude sequences below 400 bp. Mapping was performed using Minimap2 (version 2.24). Medeka (version 2.0.1) and the r1041_e82_400bps_sup_g615 model were used to map trimmed reads against the pre-consensus sequence to generate polished consensus sequences, which were manually checked for basecalling errors.

### 2.4. Phylogenetic Tree

A total of *n* = 1171 Hemagglutinin gene sequences were retrieved from NCBI and aligned with our sequence using MUSCLE (version 5.3) [34]. The Maximum-Likelihood phylogenetic analysis of Hemagglutinin gene sequences was performed by IQTREE (version 3.0.1) using the TVM + I + G4 model with 1000 bootstraps [35]. The resultant tree was then edited using iTOL (version 7.5) [36].

## 3. Results

### 3.1. Real-Time RT-PCR Screening and Sequencing

**Figure 1:**
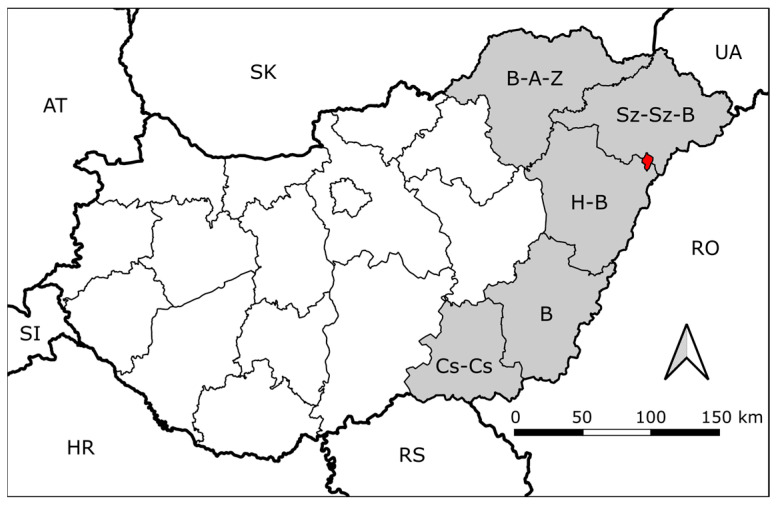
Map of Hungary showing administrative counties and neighbouring countries. Neighbouring countries are indicated using ISO country abbreviations: AT (Austria), SK (Slovakia), UA (Ukraine), RO (Romania), RS (Serbia), HR (Croatia), and SI (Slovenia). Counties included in the sampling area are highlighted in grey: B-A-Z (Borsod-Abaúj-Zemplén), Sz-Sz-B (Szabolcs-Szatmár-Bereg), H-B (Hajdú-Bihar), B (Békés), and Cs-Cs (Csongrád-Csanád). The red area indicates Nyírlugos, where the positive sample was detected.

All golden jackal samples tested negative, whereas CDV RNA was detected in both oral and rectal samples from one red fox. The animal was sampled on 29 May 2024 in the town of Nyírlugos, in Szabolcs-Szatmár-Bereg County (Figure 1). The analysis resulted in a Ct value of 28.5 for the oral swab, compared to 33.9 for the rectal swab. The oral swab was selected for sequencing due to its higher viral load.

Sequencing yielded a partial genome sequence (GenBank accession no. PX954424), 4483 nucleotides in length, comprising the 3′ end of the F gene, the complete H gene, and the 5′ end of the L gene. Due to gaps in the final sequence, only this fragment was submitted to GenBank. The coverage distribution of the sequenced genome is provided in Supplementary Materials S1.

### 3.2. Phylogenetic Analysis

**Figure 2:**
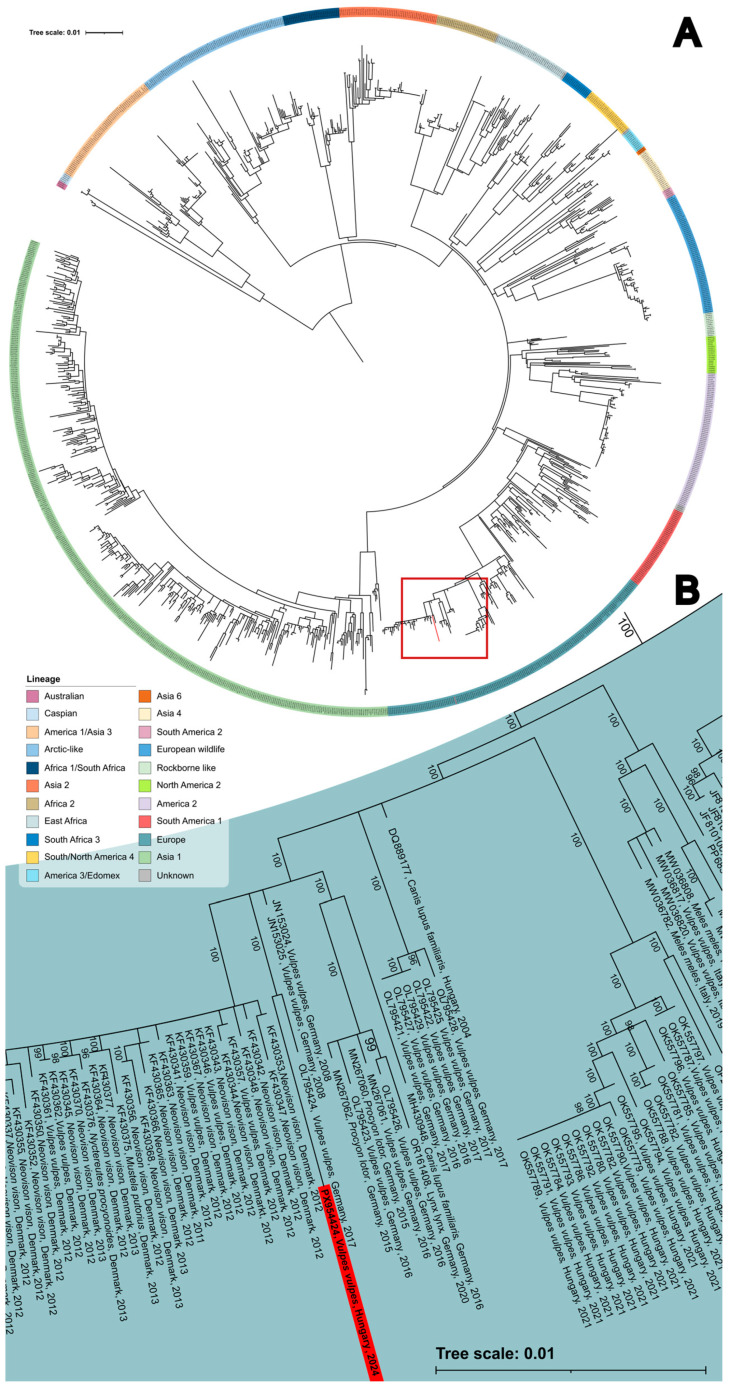
(**A**) Maximum-Likelihood phylogenetic tree based on *n* = 1171 Hemagglutinin gene nucleotide sequences. The Europe lineage is indicated in blue, with the sequence obtained in this study highlighted in red. The labels of the CDV lineages shown in the figure are displayed on the right-hand side. (**B**) Enlarged view of the Maximum-Likelihood phylogenetic tree illustrating the position of the sequence obtained in this study, indicated in red, within the Europe lineage.

Although complete genome sequencing was only partially successful due to gaps in the final sequence, the entire region of the H gene was successfully recovered, enabling phylogenetic comparison with available sequences. Based on the phylogenetic analysis of the H gene, the CDV strain was identified as a member of the Europe lineage (Figure 2). The closest relative of the identified CDV strain was a sequence obtained from a German red fox in 2017 [37]. Although the strain also clusters within the Europe lineage, together with CDV sequences detected in Hungary in 2021 [25], the phylogenetic analysis does not indicate a close genetic relationship, and the strains originate from different time periods.

## 4. Discussion

In Europe, red foxes and expanding golden jackal populations serve as reservoirs for pathogens threatening wildlife and domestic animals, and depending on the pathogen, occasionally humans. By maintaining self-sustaining infection cycles, these wild canids facilitate the spillover of zoonotic parasites (e.g., *Echinococcus multilocularis*) and bacteria (e.g., *Leptospira* and *Brucella* spp.) [38,39,40,41,42]. Regarding viral infections, while oral vaccination has largely cleared rabies from Western Europe, the virus remains endemic in the East, with Hungary serving as a critical buffer zone where it occasionally re-emerges. Furthermore, canine parvovirus [41,43] and CDV [4,44] are geographically widespread; wild canids often carry and shed high viral loads without showing immediate symptoms, posing a persistent infection risk to unvaccinated domestic dogs in shared environments [45,46,47].

The transmission of wild-canid-associated pathogens is accelerated by both biological and anthropogenic factors. These include: wildlife migration, which plays a significant role in dispersing multi-host pathogens across international borders [2,39,48,49]; urbanization and scavenging behaviours, which create opportunities for indirect contact through shared water bowls, contaminated soil, or territorial marking, providing a direct pathway for infection in domestic pets [50,51,52]; and the illegal pet trade, where the smuggling of unvaccinated puppies from Eastern Europe into the EU poses a primary risk for the reintroduction of pathogens, including unusual or non-enzootic CDV strains [53,54,55,56]. Within the specific wildlife monitoring landscape of Hungary and Central–Eastern Europe, these factors underscore the need for an integrated surveillance system to fill existing data gaps and better track pathogen circulation in wild canids.

Due to the complex epizootiology and ecology of infectious diseases in wild canids, we have initiated the development of an integrated surveillance system. This approach builds on the existing national Rabies Monitoring Program coordinated by the National Food Chain Safety Office (NÉBIH), utilizing samples collected for rabies diagnostics without requiring additional field sampling. Through collaboration between NÉBIH and the National Laboratory of Virology, molecular screening for CDV was incorporated into the established diagnostic workflow, representing an initial implementation of a cost-effective, multi-pathogen wildlife surveillance framework.

In this pilot study we demonstrated that CDV was still present among red foxes in the northeastern regions of Hungary. One of 260 red foxes tested positive for CDV by Real-Time RT-PCR, while all samples from 16 golden jackals were negative. Red foxes are well-known hosts of CDV both in Hungary and worldwide [2,25,57]. The detection of only a single positive specimen out of 260 red foxes in this study highlights the critical importance of sample size optimization in passive-surveillance programmes. Statistically, ensuring a 95% confidence level for detecting at least one positive case in a population with a low (~1%) prevalence requires a minimum of approximately 300 samples to be tested. We nearly fulfilled this requirement for red foxes, permitting us to gain insight into the circulation of CDV during the late spring–early summer period. Golden jackals are also known carriers of CDV, but the sample size for jackals in this study was below optimal [27]. A larger sample size would be necessary to draw meaningful conclusions about CDV prevalence in this population. Apparently, during outbreak situations when a higher rate of tested animals could be positive for viral RNA, even fewer than 30 samples could be sufficient to identify ongoing virus circulation in the local mesocarnivore population. For example, a study from Romania detected CDV in ~18% of fox and ~9% of jackal brain samples via viral antigen detection; this finding likely reflects a more active viral circulation in the study area [58].

Based on the H gene phylogeny, the single identified CDV was classified into the Europe lineage, which is widespread among free-ranging carnivores in Europe and poses a known risk to wildlife conservation. The study strain showed the highest similarity with a CDV detected in a red fox in 2017 from Germany [37] and clustered with other CDV strains from Germany and Denmark detected from the late 2000s to the 2020s [37,59,60]. Several cases were reported of threatened species being infected by the Europe lineage of CDV, including Eurasian lynx (*Lynx lynx*) in Germany [16] and in Switzerland [61], Iberian lynx (*Lynx pardinus)* in Spain [26], and Eurasian otter (*Lutra lutra)* [19] and Steppe polecats (*Mustela eversmanii*) in Hungary [14].

Across the continent, the Europe lineage of CDV was shown to affect multiple non-threatened species as well. In Germany, CDV was repeatedly detected from red foxes, badgers (*Meles meles*) and invasive raccoons (*Procyon lotor*) [13,37,62]. In Denmark, the same lineage infected farmed American minks (*Neovison vison)* as well as free-ranging foxes and raccoon dogs (*Nyctereutes procyonoides*) [59]. In Italy, the Europe lineage was reported in foxes, badgers and stone martens (*Martes foina*) [63,64]. In Switzerland, several foxes, badgers, stone martens and pine martens (*Martes martes*) were affected during a CDV outbreak [61]. Numerous studies showed that the Europe lineage of CDV infected not only wild-ranging mammals but also domestic dogs [28,61,65]. Thus, CDV posed a threat to wildlife conservation, and also to companion animals.

The identification of a Europe lineage CDV strain in a red fox from Nyírlugos indicates a potential role of transboundary wildlife movement in the regional circulation of CDV, given the proximity of the sampling site to the Romanian and Ukrainian borders. However, the current absence of publicly available CDV sequences from wildlife in Romania and Ukraine, as well as the limited molecular characterization of strains circulating in local Hungarian companion animals, precludes definitive tracing of the infection route or confirmation of these suspected transboundary links. The genetic similarity of the identified strain to CDV strains identified in Germany and Denmark suggests that this lineage may be widely but sporadically distributed across the continent. This underscores the critical need for a more comprehensive and integrated genomic surveillance approach to identify co-circulating pathogens and bridge the data gaps between domestic- and wild-animal health.

The study is limited by the inability to obtain a complete genome sequence, which was most likely due to sample quality, genome fragmentation and restricted phylogenetic analyses to the H gene region. Clinical information for the sampled animal was unavailable, preventing documentation of any associated clinical signs. Moreover, CDV detection was based on a single positive sample, limiting broader epidemiological inferences. Nevertheless, the study provides valuable molecular data from a previously underrepresented region and contributes to the understanding of CDV genetic diversity in Europe.

## 5. Conclusions

The broad host range of CDV highlights the need for further research to better understand the mechanisms underlying its transmission and pathogenesis. Surveillance using integrated approaches provides valuable information on the circulation and distribution of pathogens in the region, thereby supporting wildlife conservation and outbreak prevention. As this study confirms, the availability of genetic data is crucial for understanding viruses of epidemiological importance. In our study, we aimed to demonstrate, with an integrated surveillance system, a new approach in the region, showing that virus monitoring can be achieved without launching separate programmes and that the technological background is available for effective genomic surveillance, considering that scaling the system may involve practical challenges such as funding, laboratory standardization, and data sharing.

## Figures and Tables

**Table 1:** County-level distribution of the number of examined individuals included in the study.

County	Red Fox (*n*)	Golden Jackal (*n*)
Békés	60	5
Borsod-Abaúj-Zemplén	77	3
Csongrád-Csanád	5	1
Hajdú-Bihar	24	3
Szabolcs-Szatmár-Bereg	94	4
Sum	260	16

## Data Availability

The sequence generated has been deposited in the NCBI GenBank database with accession number PX954424. The datasets generated during and/or analysed during the current study are available from the corresponding author upon request.

## Supplementary Materials

The following supporting information can be downloaded at: https://www.mdpi.com/article/10.3390/v18030352/s1, Figure S1: Visualization of sequencing coverage obtained using the amplicon-based sequencing method.

## Abbreviations

The following abbreviations are used in this manuscript:

cDNA	Complementary deoxyribonucleic acid
CDV	Canine distemper virus
dsDNA	Double-stranded deoxyribonucleic acid
H gene	Hemagglutinin gene
H protein	Hemagglutinin protein
NCBI	National Center for Biotechnology Information
NEB	New England Biolabs
NGS	Next-generation sequencing
NÉBIH	National Food Chain Safety Office
PBS	Phosphate buffered-saline
PCR	Polymerase chain reaction
RT-PCR	Reverse-Transcription Polymerase Chain Reaction
RT-qPCR	Reverse-Transcription Quantitative Polymerase Chain Reaction
