# Supplementary material for: Detection and Phylogenetic Characterization of Canine Distemper Virus from a Red Fox in Hungary
Source: Viruses. 2026 Mar 13;18(3):352. doi: 10.3390/v18030352 (PMC13030248; doi:10.3390/v18030352)
Supplement: Supplementary file 1 [file viruses-18-00352-s001.zip › Supplementary Materials S1.pdf]

viruses

MDPI

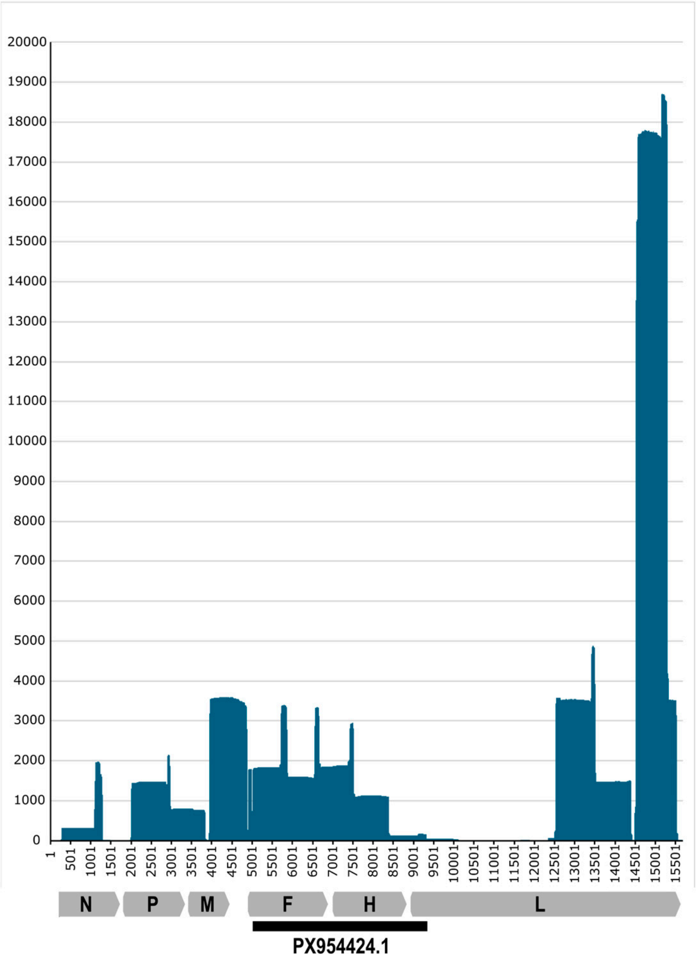

Supplementary Materials S1: Visualization of sequencing coverage obtained using the amplicon-based sequencing method. Horizontal scale represents the genomic position, whilst the vertical scale displays the coverage values of the sequencing reaction. The black line at the bottom of the figure represents the sequence deposited in GenBank.
